# Heterogeneity of Ocular Hemodynamic Biomarkers among Open Angle Glaucoma Patients of African and European Descent

**DOI:** 10.3390/jcm12041287

**Published:** 2023-02-06

**Authors:** Brent Siesky, Alon Harris, Alice Verticchio Vercellin, Julia Arciero, Brendan Fry, George Eckert, Giovanna Guidoboni, Francesco Oddone, Gal Antman

**Affiliations:** 1 Department of Ophthalmology, Icahn School of Medicine at Mount Sinai, New York, NY 10029, USA; 2 Department of Mathematical Sciences, Indiana University-Purdue University Indianapolis, Indianapolis, IN 46202, USA; 3 Department of Mathematics and Statistics, Metropolitan State University of Denver, Denver, CO 80217, USA; 4 Department of Biostatistics, Indiana University School of Medicine, Indianapolis, IN 46202, USA; 5 Department of Electrical Engineering and Computer Science, University of Missouri, Columbia, MO 65211, USA; 6 IRCCS Fondazione Bietti, 00198 Rome, Italy; 7 Department of Ophthalmology, Rabin Medical Center, Petach Tikwa 4941492, Israel

**Keywords:** open angle glaucoma, risk factors, optical coherence tomography angiography, Asian descent, African descent, Hispanic descent, European descent

## Abstract

This study investigated the heterogeneity of ocular hemodynamic biomarkers in early open angle glaucoma (OAG) patients and healthy controls of African (AD) and European descent (ED). Sixty OAG patients (38 ED, 22 AD) and 65 healthy controls (47 ED, 18 AD) participated in a prospective, cross-sectional study assessing: intraocular pressure (IOP), blood pressure (BP), ocular perfusion pressure (OPP), visual field (VF) and vascular densities (VD) via optical coherence tomography angiography (OCTA). Comparisons between outcomes were adjusted for age, diabetes status and BP. VF, IOP, BP and OPP were not significantly different between OAG subgroups or controls. Multiple VD biomarkers were significantly lower in OAG patients of ED (*p* < 0.05) while central macular VD was lower in OAG patients of AD vs. OAG patients of ED (*p* = 0.024). Macular and parafoveal thickness were significantly lower in AD OAG patients compared to those of ED (*p* = 0.006–0.049). OAG patients of AD had a negative correlation between IOP and VF index (r = −0.86) while ED patients had a slightly positive relationship (r = 0.26); difference between groups (*p* < 0.001). Age-adjusted OCTA biomarkers exhibit significant variation in early OAG patients of AD and ED.

## 1. Introduction

The onset and progression of open angle glaucoma (OAG) in many persons, despite significant intraocular pressure (IOP) reduction, motivates the need for evolved approaches to individualize patient care. Among IOP-independent risk factors, impaired vascular regulation and ocular tissue ischemia have been well documented [1]. Over time, technological advancements in optical imaging have allowed for more specific and detailed quantification of various vascular elements within the eye. Translating these diverse vascular biomarkers into an effective tool to assist clinicians in glaucoma management, however, remains a significant challenge due to their inaccessibility and complexity in analysis and clinical application. Precision in ocular vascular data is also often lacking, with many custom imaging modalities and analysis approaches producing unique, limited and often difficult-to-reproduce aspects of ocular hemodynamics and metabolism [1]. In addition, retinal vascular network heterogeneity across glaucomatous populations is relatively unknown and shared vascular comorbidities including hypertension and diabetes are significantly higher in certain demographic groups, all of which may alter vascular imaging biomarkers and overall risk modeling. Further, differences in tissue pigmentation among subjects may alter or prohibit optical imaging biomarker comparability.

Optical coherence tomography angiography (OCTA) allows simultaneous assessments of retinal and optic nerve head (ONH) structure and vascular densities (VD), providing clinicians an improved user interface for the potential inclusion of vascular biomarkers [2]. Since its introduction, OCTA data from a wide range of studies have suggested a significant reduction in the mean peripapillary VD in glaucoma patients, as well as marked reduction in VD in the ONH and parafoveal regions [3]. The associations of OCTA VD outcomes and OAG are generally strong, suggesting lower VD of the peripapillary retina, ONH and macular regions in glaucoma patients when compared to healthy controls [2,3]. A recent study in a Chinese population found that significant microvascular damage assessed by OCTA was present in both macular and peripapillary regions in early OAG patients, with VD loss highest in peripheral regions [4]. Despite these demonstrated associations of OCTA VD biomarkers and OAG, there is still a lack of understanding of retina, macula and ONH VD loss in early glaucomatous disease. Further, little is known regarding retinal vascular networks and associated biomarkers across different patient populations, limiting comparative analysis of the earliest microvascular changes in OAG prior to detectible visual field loss.

OCTA VD biomarkers have demonstrated acceptable test–retest variability as well as the potential to differentiate glaucomatous from normal eyes [5]. However, OCTA biomarker data and truncated normative data available for cross-subject analysis may not be robust enough to ensure sufficient rigor for all demographic comparisons. The African American Eye Disease Study found age, sex and diabetes status must be considered when assessing changes in radial peripapillary capillary VD in glaucoma [6]. While identifying consistently lower VD in glaucoma patients across multiple studies, a recent study also concluded that a well-controlled meta-analysis of OCTA and glaucoma was not possible due to the wide variation in utilized methods, measurement approaches and selected regions of VD [7]. Therefore, although evidence supporting OCTA biomarker utility in OAG is fairly strong, the unknown heterogeneity of OCTA data and lack of population-based longitudinal studies currently limits its full applicability.

The principles of operation for OCTA involve utilization of ocular structures, light dynamics and tissue pigment levels, all of which may differ between persons with OAG and therefore bias device outcomes [1,2,3,5,7,8,9]. Mitigating potential bias in ophthalmic diagnostic utilities is essential to reduce disparities in treatment outcomes currently experienced by OAG patients of African descent (AD) [10]. Specifically, a broad array of cross-sectional VD data is available in Asian descent and European descent (ED) populations, but little to no comparative data is available on OCTA VD biomarkers in early OAG patients of AD who may have higher vascular impairment in their disease process [11]. This pilot study therefore first investigates the heterogeneity and variability in OCTA-assessed retinal, ONH and macular VD and ocular structure biomarkers and then analyzes their relationships to IOP, blood pressure (BP) and ocular perfusion pressures (OPP) in early stage OAG patients of AD and ED and age-adjusted healthy controls.

## 2. Materials and Methods

Sixty OAG patients (38 ED, 22 AD) exhibiting early structural glaucomatous changes and 65 healthy controls (47 ED, 18 AD) participated in a prospective, cross-sectional, observational study conducted at the Icahn School of Medicine at Mount Sinai, New York, NY. Healthy subjects were free from OAG and any other eye disease (i.e., age-related macular degeneration, diabetic retinopathy) while OAG subjects only had OAG disease as confirmed by a board-certified glaucoma specialist. OAG patients with early structural glaucomatous changes presented evidence of the following: ONH or RNFL structural abnormalities as defined by the 2020 American Academy of Ophthalmology Primary Open-Angle Glaucoma Preferred Practice pattern [12]: diffuse or focal narrowing, or notching, of the optic disc rim, especially at the inferior or superior poles; progressive narrowing of the neuro-retinal rim with an associated increase in cupping of the optic disc; diffuse or localized thinning of the parapapillary RNFL, especially at the inferior or superior poles; optic disc hemorrhages involving the disc rim, parapapillary RNFL, or lamina cribrosa; optic disc neural rim asymmetry of the two eyes consistent with loss of neural tissue; beta-zone parapapillary atrophy; thinning of the RNFL and/or macula on imaging. The study was conducted in accordance with the Declaration of Helsinki and approved by the Institutional Review Board of Icahn School of Medicine at Mount Sinai, New York, NY (protocol code: Study-20-00198; date of approval: 17 November 2021). Written informed consent was obtained from all subjects involved in the study.

All participants were required to meet the following inclusion criteria: age 21 years or older, healthy eyes without OAG and any other eye disease and/ or patients with confirmed early OAG in at least one eye by a glaucoma specialist with no other eye disease. Patients were excluded for the following reasons: refractive error >+9 Diopters and <−9 Diopters in spherical equivalent; evidence of exfoliation or pigment dispersion; eye disease other than glaucoma or other eye health concerns; use of eye medications (other than IOP lowering medications for glaucoma or eye lubricants for dry eye); neurological disease (Alzheimer’s disease, Parkinson’s disease, multiple sclerosis); psychosis or neurological diseases that could prevent reliable eye exams; severe, unstable or uncontrolled cardiovascular, renal, or pulmonary disease.

One qualified study eye was randomly (coin flip) selected for each subject and patients were seen during a single two-hour study visit undergoing assessment for: IOP via Goldmann applanation, systolic and diastolic blood pressure (BP) (ambulatory, at rest), ocular perfusion pressure (OPP = 2/3*mean arterial pressure-IOP) and visual field index (VFI), mean deviation (MD) and pattern standard deviation (PSD) via Humphrey field analyzer II using the 24-2 Swedish interactive threshold algorithm standard (white III stimulus) V.4.1 (Carl Zeiss Mediatec, Dublin, CA, USA).

OCTA (Optovue Inc, Avanti Angiovue, Fremont, CA, USA) was used to acquire structural elements, including RNFL thickness, cup-to-disc (C/D) ratio, ganglion cell complex (GCC) thickness and VD of the retina, ONH and macular regions. In brief, the Optovue OCTA provides a non-invasive three-dimensional visualization of the retinal microvasculature and OCT-assessed structural elements of the retina, ONH and macula regions. The device uses consecutive scans to calculate motion contrast and translates initial tissue reflectance into flow signals; details on principles of OCTA are available elsewhere [5,8,9]. In our study, we utilized the AngioAnalytics^TM^ licensed upgrade present in the Avanti Optovue device that enables the automatic assessment of the retinal and ONH VD computed as percentage of area occupied by OCTA detected vasculature in the areas of interest [13]. In detail, the ONH VD were assessed via the 4.5 mm HD Angio Disc scan and the percentage of area occupied by OCTA detected vasculature was detected for the radial peripapillary capillary (RPC) slab (from the internal limiting membrane to the nerve fiber layer). The ONH vessel density measurements are provided from three regions: peripapillary region (defined by two rings of 2 mm and 4 mm centered on disc center); inside the optic disc; entire region. The VD are measured for the small vessels (SV) (i.e., with large vessel masking) and for all vessels (ALL); the application of large vessel mask has threshold of ≥3 pixels (approximately ≥33 μm). The ONH vessels density information included were the RPC slab density parameters (global and peripapillary hemispheric for both the small vessels and all vessels) and the regional vessel density parameters (small vessels only) for the superior, temporal, nasal and inferior quadrants). The central macular vessel density was assessed via the 6.0 mm HD AngioRetina scan in the 1-mm central ring of the ETDRS grid centered on the fovea [13].

Healthy subjects were free from eye disease other than mild myopia and well-controlled hypertension. Subject demographics, including age, self-reported race and sex, height, weight, diabetic status, hypertensive status and medication use, were also recorded.

Nonparametric analysis of covariance was used to compare race/ethnic groups in OAG patients and healthy controls for differences in biomarkers, with demographics as covariates in the analyses including sex, age, hypertension and diabetes. Spearman correlations were used to test associations between biomarkers, with adjustment for demographics. A 5% significance level was used. Statistical analyses were performed using SAS (SAS Institute Inc., Cary, NC, USA).

## 3. Results

Ocular and systemic characteristics for OAG patients and healthy controls are shown in Table 1. VF parameters, IOP, BP and OPP were not significantly different (*p* > 0.05) between OAG or healthy subgroups except for BP systolic between OAG patients and controls of ED (*p* = 0.000) and VF PSD between OAG patients and controls of AD (*p* = 0.036), as showed in Table 1.

When assessing ocular structure outcomes between heathy and OAG patients, average RNFL thickness was identified to be significantly thinner in early OAG patients of AD (*p* = 0.007) and ED (*p* = 0.000) as shown in Table 2. RNFL thickness in the superior and inferior hemispheres were also statistically significantly different between OAG patient groups and controls (*p* < 0.05). C/D area ratio (*p* = 0.023) and disc area (*p* = 0.028) were significantly different in OAG patients of ED versus controls.

Macular and parafoveal thicknesses were significantly lower in AD OAG patients compared to those of ED (*p* = 0.006–0.049). No other significant differences were found in the structure of the macular regions for OAG patient groups or between OAG patients and controls.

Multiple radial peripapillary capillary VD biomarkers were significantly lower only in OAG patients of ED compared to controls (*p* = 0.000–0.013) while central macular VD was lower only in OAG patients of AD compared to OAG patients of ED (*p* = 0.024). Table 3 show the OCTA parameters in OAG patients and healthy subjects of AD and ED.

In examination of the relationships between biomarkers, several significant differences in correlations were found between OAG patients of AD versus OAG patients of ED (Table 4). Regarding the relationship between IOP and VF parameters, OAG patients of AD had a negative correlation between IOP and VF index (r = −0.86) while ED patients had a slightly positive relationship (r = 0.26) leading to a statistically significant difference between groups (*p* < 0.001), see Table 4.

The differences in correlations between IOP and structural or VD outcomes for any control groups of AD and ED are shown in Table 5.

## 4. Discussion

This pilot analysis examined the heterogeneity of OCTA hemodynamic biomarkers of VD in the retina, macula and ONH in early stage OAG patients exhibiting structural changes and healthy controls of AD and ED. While a wealth of data supports OCTA-measured VD biomarkers being associated with OAG, little data is currently available on VD loss in early stage OAG across differing population groups. Early capillary loss prior to detectible VF damage may indicate a primary vascular dysfunction in the OAG disease process and such impaired vascular regulation may be more prevalent in persons of AD [11,14]. The shared and elevated rates of vascular comorbidities in AD populations may indicate higher vascular involvement in the glaucomatous disease process and OCTA VD may therefore have higher utility in AD disease management compared to other population groups.

Understanding variability within populations is especially important since glaucoma is multifactorial and may represent a collection of diseases that involve higher vascular insult in certain persons. Specifically, persons of AD are known to have significantly higher rates of systemic vascular health disease, including stroke, hypertension and diabetes [15,16] and these translate into poor autoregulatory ability for microvascular tissues. We previously identified retinal and retrobulbar blood flow deficits in glaucoma patients of AD compared to those of ED [14] and linked these lower vascular biomarkers to elevated levels of structural disease progression in AD OAG patents over a four-year period [11]. Of significant importance, it is not currently known if OAG patients with higher systemic vascular disease experience more significant retinal, macular and/or ONH VD capillary prior to detectable visual field loss. In addition, scarce information is currently available on OCTA VD biomarkers in persons of Hispanic descent, who along with persons of AD may experience elevated levels of both systemic vascular disease and glaucomatous disease burden. As these and other imaging biomarkers are utilized in clinical management, understanding bias in access, utilization and application of data is critical to eliminate bias and subsequent OAG disease and treatment disparities, including those currently experienced by patients of AD [10].

In our pilot analysis, IOP, BP and OPP were not significantly different between OAG or healthy subgroups. VF outcomes were also not different between OAG subgroups or OAG and controls, with the exception of VF PSD between OAG patients (3.24) and controls (2.10) of AD (*p* = 0.036). These data confirm our OAG groups (based upon changes to the ONH and retina) were relatively early in stage and examined prior to significant perimetric defect(s) and significant tissue atrophy. Specifically, average RNFL thickness was significantly lower in OAG patients of AD and ED compared to controls. Superior and inferior hemispheres RNFL thickness were also statistically significantly different between OAG patient groups and controls. Average GCC thickness was significantly lower in OAG patients of AD versus controls (80.1 vs 93.1) while trending (non-significantly) lower in those of ED (mean: 88.5 vs 95.3). A larger sample size would increase statistical power and likely result in more significant differences in each group, although the power of sensitivity for OCTA RNFL thickness and GCC thickness appears to vary with higher sensitivity in OAG patients of ED. It is important to note, however, that determining OAG early prior to significant visual field loss is challenging and lack of exact uniformity in early OAG structural changes may limit applicability of comparisons to other groups.

Macular and parafoveal thickness were significantly lower in AD OAG patients compared to those of ED. This finding in early AD OAG patients is interesting as early OAG structural changes have been previously reported in pilot work via OCTA that include reduction in the superficial VD of the macular regions [5]. This pilot data may indicate OAG patients of AD exhibit earlier, vascular-related changes to the macular region of the eye prior to or just at detectable visual field loss. Improvement in modeling outcomes for OAG patients where VD and structure are combined has been previously reported utilizing specific OCTA VD biomarkers [17]. Taken together with earlier pilot data [11,14], these data suggest modeling for OAG risk in persons of AD should include vascular elements where possible, including assessment of macular regions in early OAG.

Multiple VD biomarkers were significantly lower in OAG patients of ED while central macular VD was lower only in OAG patients of AD. This suggests OCTA VD biomarkers, similar to structural elements in the study, may have higher sensitivity in discriminating early OAG patients of ED than in those of AD. This may be due to more robust use of ED eyes for OCTA development compared to those of AD or a result of elevated levels of pigmentation altering light-based OCTA comparative outcomes. Differences in pigmentation of tissue affecting ocular imagery is an established limitation of optical imaging modalities including photographic retinal oximetry, where retinal oxygen levels often exceed 100%, including in persons of AD with higher levels of pigmentation [1,18].

Interestingly, our exploratory analysis showed that OAG patients of AD had a negative correlation between IOP and VF index (r = -0.86) compared to those of ED, who had a slightly positive relationship (r = 0.26), with a significant difference between groups (*p* < 0.001). While pilot data on differences in ocular structure, IOP, disease burden and therapeutic outcomes between OAG patients of AD and ED are available [10], little is known about differences in the relationships between risk factors. The strong negative relationship seen in our data between VFI and IOP in OAG subjects of AD agrees with several previous studies reporting higher IOP levels associated with increased OAG disease persons of AD [10]. Previously, higher levels of vascular dysfunction were also found in OAG patents of AD compared to ED, regardless of similar IOP and disease status [11,14]. Together these data suggest that OAG patients have both IOP and significant vascular mechanisms involved in their disease process, with some level of variability. While a higher sample size may reduce the magnitude of observed differences, these data suggest that traditional risk metrics using IOP alone may not best capture risk for all individuals of AD and ED. Differences in relationships between physiological biomarkers may also help explain why similar therapeutic IOP reduction results in significantly difference disease and visual field outcomes [10] for OAG patients of AD. Together, these data point to the need to model for multi-input risk in OAG to account for differential risk and individualization in disease management plans tailored to preservation of visual function and not considering only the level of IOP reduction achieved.

Our pilot study has several significant limitations to acknowledge. First, our patient population was considered early-stage OAG in nature; the structural diagnostic elements (ONH, CD ratio, RNFL) and diabetic and/or hypertensive status were not uniform across all subjects. These differences in potential approach and difficulty in consensus of determining early glaucoma may also limit applicability of comparisons to other patient samples. While out study was indented to be pilot in nature, the sample size, while informative, is small and a larger sample would likely reduce the magnitude of observed differences. In addition, healthy subjects were significantly younger than OAG subjects, although results were statistically adjusted for age to limit impact on the samples. Finally, our analysis was cross-sectional; longitudinal glaucomatous changes in outcomes were not assessed and are required for determining influence on predictability of disease progression.

In this analysis, age-adjusted OCTA hemodynamic and structural factors exhibit significant variation in OAG patients of AD and ED. For any measured outcome, use of normative data and disease group comparisons require careful consideration of potential biases including: limited access, non-inclusive principles of device operation (i.e., not accounting for differences in patient pigmentation) and bias in data utilization and modeling. To uncover the best approach for each patient, an inclusive model of risk is needed that accounts for potential modifiers and bias in patient data. Data rigor from ocular imaging is important to address in order to reduce disparities in current and future applications. Efforts to account for heterogeneity in OCTA hemodynamic data may be especially important in persons with elevated vascular disease including persons of AD. Properly designed longitudinal studies that target AD outcomes as primary endpoints are needed to confirm these findings and understand the impact of OCTA biomarker heterogeneity on glaucoma progression and disease management.

## Figures and Tables

**Table 1:** Mean and standard deviation of ocular and systemic parameters in control and open-angle glaucoma (OAG) patients of African descent (AD) and European descent (ED). Comparisons (*p*-values) between outcomes were adjusted for age, diabetes status and BP. BP: blood pressure; bpm: beats per minute; HR: heart rate; IOP: intraocular pressure; MAP: mean arterial pressure; MD: mean deviation; OPP: ocular perfusion pressure; PSD: pattern standard deviation; VF: visual field; VFI: visual field index. * *p* < 0.005.

	Patients Groups	AD	*p*-Value AD Healthy vs. OAG	ED	*p*-Value ED Healthy vs. OAG
IOP (mmHg)	Control	14.44 (2.38)	0.556	14.17 (2.72)	0.978
	OAG	16.32 (4.03)		16.83 (5.31)	
BP Systolic (mmHg)	Control	126.94 (21.06)	0.135	116.85 (13.64)	0.000 *
	OAG	135.86 (21.84)		131.22 (14.18)	
BP Diastolic (mmHg)	Control	74.94 (13.45)	0.121	76.85 (10.02)	0.686
	OAG	82.09 (13.89)		76.17 (9.94)	
HR (bpm)	Control	75.18 (15.46)	0.660	70.25 (11.13)	0.277
	OAG	73.41 (14.64)		67.94 (10.76)	
MAP (mmHg)	Control	92.28 (14.76)	0.070	90.19 (10.39)	0.060
	OAG	99.95 (14.30)		94.53 (9.89)	
OPP (mmHg)	Control	47.06 (10.14)	0.365	45.87 (6.72)	0.446
	OAG	50.32 (10.71)		46.44 (8.08)	
VF MD (decibel)	Control	−1.43 (1.72)	0.204	−1.43 (1.88)	0.537
	OAG	−2.73 (1.96)		−2.34 (3.42)	
VF PSD (decibel)	Control	2.10 (0.74)	0.036 *	2.19 (2.25)	0.484
	OAG	3.24 (1.69)		3.38 (2.14)	
VF VFI (%)	Control	97.45 (2.62)	0.247	98.05 (4.06)	0.191
	OAG	95.07 (3.87)		94.11 (7.87)	

**Table 2:** Mean and standard deviation of the optical coherence tomography parameters and comparisons (*p*-values) in control and open-angle glaucoma (OAG) patients of African descent (AD) and European descent (ED). C/D: cup-to-disc; GCC: ganglion cell complex thickness; H: horizontal; RNFL: Retinal Nerve Fiber Layer thickness; V: vertical. * *p* < 0.005.

	Patients Group	AD	*p*-Value AD Healthy vs. OAG	ED	*p*-Value ED Healthy vs. OAG
Average RNFL (µm)	Control	101.07 (12.46)	0.007 *	101.19 (7.94)	0.000 *
	OAG	79.86 (14.75)		79.88 (10.78)	
Superior RNFL (µm)	Control	102.73 (15.27)	0.023 *	102.83 (9.55)	0.000 *
	OAG	81.14 (14.86)		81.69 (12.04)	
Inferior RNFL (µm)	Control	99.47 (10.73)	0.018 *	99.60 (7.32)	0.000 *
	OAG	78.71 (15.85)		77.78 (12.27)	
C/D Area Ratio	Control	0.40 (0.13)	0.160	0.25 (0.13)	0.023 *
	OAG	0.58 (0.13)		0.52 (0.19)	
C/D V Ratio	Control	0.55 (0.15)	0.934	0.42 (0.17)	0.137
	OAG	0.76 (0.12)		0.70 (0.17)	
C/D H Ratio	Control	0.62 (0.19)	0.934	0.54 (0.19)	0.303
	OAG	0.80 (0.10)		0.75 (0.16)	
Rim area (mm^2^)	Control	1.45 (0.29)	0.917	1.41 (0.26)	0.930
	OAG	0.86 (0.22)		0.84 (0.28)	
Disc area (mm^2^)	Control	2.28 (0.44)	0.923	1.87 (0.29)	0.028 *
	OAG	2.12 (0.33)		1.80 (0.42)	
Cup Volume (mm^3^)	Control	0.22 (0.17)	0.116	0.09 (0.10)	0.871
	OAG	0.47 (0.35)		0.27 (0.23)	
Average GCC (µm)	Control	93.13 (8.72)	0.037 *	95.30 (7.57)	0.120
	OAG	80.09 (12.47)		88.54 (19.07)	

**Table 3:** Mean and standard deviation of the optical coherence tomography angiography (OCTA) vessel density (VD) and comparisons (*p*-values) in control and open-angle glaucoma (OAG) patients of African descent (AD) and European descent (ED). The VD represents the percentage (%) of area occupied by OCTA detected vasculature. The optic nerve head (ONH) VD were assessed via the 4.5 mm HD Angio Disc scan and the percentage of area occupied by OCTA detected vasculature was detected for radial peripapillary capillary slab (from the internal limiting membrane to the nerve fiber layer). The ONH VD measurements are provided from three regions: peripapillary region (defined by two rings of 2 mm and 4mm centered on disc center); inside the optic disc (“Inside Disc”); and entire region (“Whole Image”). The VD are measured for the small vessels (SV) (i.e., with large vessel masking) and for all vessels (ALL). The vessels density information include the radial peripapillary capillary slab density parameters (global and peripapillary hemispheric for both the small vessels and all vessels) and the regional peripapillary vessel density parameters (small vessels only) for the superior, temporal, nasal and inferior quadrants. The central macular vessel density was assessed via the 6.0 mm HD AngioRetina scan in the 1-mm central ring of the ETDRS grid centered on the fovea. ALL: all vessels; IH: inferior hemisphere; IQ: inferior quadrant; NQ: nasal quadrant; RPC: radial peripapillary capillary; SH: superior hemisphere; SQ: superior quadrant; SV: small vessels; TQ: temporal quadrant. * *p* < 0.005.

	Patients Group	AD	*p*-Value AD Heathy vs. OAG	ED	*p*-Value ED Heathy vs. OAG
Optic nerve head vessel density in the radial peripapillary capillary (RPC) slab					
Whole Image SV (%)	Control	49.28 (2.86)	0.528	49.48 (2.05)	0.001 *
	OAG	44.93 (3.63)		43.27 (4.91)	
Inside Disc SV (%)	Control	48.51 (6.01)	0.598	50.00 (6.01)	0.505
	OAG	45.46 (6.15)		48.96 (8.15)	
Peripapillary SV global (%)	Control	51.53 (4.67)	0.540	52.28 (2.10)	0.001 *
	OAG	46.36 (5.77)		44.98 (6.42)	
Peripapillary SV in the SH (%)	Control	51.51 (5.35)	0.833	52.58 (2.51)	0.000 *
	OAG	46.17 (6.18)		44.17 (9.76)	
Peripapillary SV in the IH (%)	Control	51.55 (4.18)	0.973	51.94 (2.23)	0.008 *
	OAG	46.54 (6.33)		43.99 (7.44)	
Whole Image ALL (%)	Control	55.43 (2.95)	0.197	55.76 (2.45)	0.000 *
	OAG	50.51 (3.64)		48.71 (5.06)	
Inside Disc ALL (%)	Control	91.40(128.55)	0.039 *	59.34 (5.35)	0.962
	OAG	54.31 (5.10)		56.36 (6.95)	
Peripapillary ALL global (%)	Control	57.50 (4.47)	0.355	58.15 (2.46)	0.000 *
	OAG	51.62 (5.70)		48.85 (9.99)	
Peripapillary ALL in the SH (%)	Control	57.85 (5.25)	0.525	58.80 (2.50)	0.000*
	OAG	51.71 (6.09)		50.89 (6.68)	
Peripapillary ALL in the IH (%)	Control	57.09 (3.91)	0.634	57.61 (2.63)	0.013 *
	OAG	51.60 (6.09)		49.39 (7.12)	
Peripapillary SV in the SQ (%)	Control	51.73 (5.15)	0.403	53.18 (3.19)	0.005 *
	OAG	45.30 (7.41)		44.79 (7.34)	
Peripapillary SV in the NQ (%)	Control	50.38 (4.27)	0.089	49.41 (3.97)	0.001 *
	OAG	43.05 (6.00)		40.91 (7.23)	
Peripapillary SV in the IQ (%)	Control	54.47 (4.66)	0.163	53.97 (2.66)	0.004 *
	OAG	47.45 (6.05)		46.32 (7.23)	
Peripapillary SV in the TQ (%)	Control	52.40 (4.53)	0.037 *	53.94 (3.29)	0.655
	OAG	50.45 (8.02)		49.47 (8.36)	
Central Macular vessel density (%)	Control	13.40 (8.09)	0.274	21.76 (6.85)	0.482
	OAG	15.47 (8.71)		19.45 (7.42)	

**Table 4:** Spearman correlations (r) between intraocular pressure (IOP) and functional, structural and hemodynamic biomarkers in open-angle glaucoma patients of African descent (AD) and European descent (ED). ALL: all vessels; C/D: cup-to-disc; GCC: ganglion cell complex thickness; IH: inferior hemisphere; IQ: inferior quadrant; MD: mean deviation; NQ: nasal quadrant; PSD: pattern standard deviation; RNFL: Retinal Nerve Fiber Layer thickness; RPC: radial peripapillary capillary; SH: superior hemisphere; SQ: superior quadrant; SV: small vessels; TQ: temporal quadrant; VD: vessel density(%); VF: visual field; VFI: visual field index. * *p* < 0.005.

	OAG AD		OAG ED		AD vs. ED
Correlation between IOP (mmHg) with	r	*p*	r	*p*	*p*
VF MD (decibel)	−0.24	0.553	0.15	0.578	0.282
VF PSD (decibel)	0.22	0.585	−0.39	0.136	0.084
VF VFI (%)	−0.86	0.004 *	0.26	0.358	0.000 *
Average RNFL (µm)	−0.40	0.166	0.49	0.045 *	0.004 *
Superior RNFL (µm)	−0.32	0.278	0.29	0.270	0.059
Inferior RNFL (µm)	−0.37	0.193	0.59	0.012 *	0.001 *
C/D Area Ratio	0.34	0.261	−0.60	0.009 *	0.002 *
C/D Vertical Ratio	0.27	0.384	−0.60	0.010 *	0.004 *
C/D Horizontal Ratio	0.16	0.599	−0.42	0.090	0.065
Rim area (mm^2^)	−0.16	0.605	0.41	0.104	0.074
Disc area (mm^2^)	0.42	0.151	−0.47	0.059	0.004 *
Cup Volume (mm^3^)	0.16	0.604	−0.48	0.053	0.042 *
Average GCC (µm)	−0.30	0.289	0.20	0.381	0.101
VD RPC Whole Image SV (%)	−0.15	0.637	0.39	0.101	0.089
VD RPC Inside Disc SV (%)	0.14	0.657	−0.01	0.970	0.648
VD RPC Peripapillary global SV (%)	−0.17	0.578	0.32	0.181	0.120
VD RPC Peripapillary in the SH SV (%)	−0.30	0.330	0.25	0.304	0.086
VD RPC Peripapillary in the IH SV (%)	0.04	0.903	0.38	0.106	0.265
VD RPC Whole Image ALL (%)	−0.28	0.365	0.47	0.040 *	0.015 *
VD RPC Inside Disc ALL (%)	0.18	0.554	0.04	0.873	0.655
VD RPC Peripapillary global ALL (%)	−0.29	0.354	0.21	0.393	0.123
VD RPC Peripapillary in the SH ALL (%)	−0.41	0.168	0.27	0.271	0.031 *
VD RPC Peripapillary in the IH ALL (%)	−0.01	0.968	0.39	0.095	0.191
VD RPC Peripapillary in the SQ SV (%)	−0.30	0.333	−0.06	0.795	0.463
VD RPC Peripapillary in the NQ SV (%)	−0.32	0.299	0.24	0.324	0.080
VD RPC Peripapillary in the IQ SV (%)	−0.05	0.865	0.25	0.326	0.354
VD RPC Peripapillary in the TQ SV (%)	0.13	0.688	0.29	0.240	0.611
VD Central Macular (%)	−0.09	0.779	−0.08	0.736	0.978

**Table 5:** Spearman correlations (r) between intraocular pressure (IOP) and functional, structural and hemodynamic biomarkers in controls subjects of African descent (AD) and European descent (ED). ALL: all vessels; C/D: cup-to-disc; GCC: ganglion cell complex thickness; IH: inferior hemisphere; IQ: inferior quadrant; MD: mean deviation; NQ: nasal quadrant; PSD: pattern standard deviation; RNFL: Retinal Nerve Fiber Layer thickness (µm); RPC: radial peripapillary capillary; SH: superior hemisphere; SQ: superior quadrant; SV: small vessels; TQ: temporal quadrant; VD: vessel density; VF: visual field; VFI: visual field index. * *p* < 0.005.

	Healthy AD		Healthy ED		AD vs. ED
Correlation between IOP (mmHg) with	r	*p*	r	*p*	*p*
VF MD (decibel)	−0.58	0.344	−0.25	0.149	0.322
VF PSD (decibel)	0.93	0.018 *	−0.18	0.312	0.000 *
VF VFI (%)	−0.62	0.309	0.10	0.576	0.047 *
Average RNFL (µm)	−0.54	0.299	−0.23	0.188	0.347
Superior RNFL (µm)	−0.58	0.254	−0.21	0.226	0.255
Inferior RNFL (µm)	−0.56	0.276	−0.17	0.345 *	0.236
C/D Area Ratio	0.08	0.891	−0.14	0.435	0.571
C/D Vertical Ratio	0.13	0.827	−0.06	0.750	0.638
C/D Horizontal Ratio	0.24	0.677	−0.14	0.449	0.334
Rim area (mm^2^)	−0.53	0.309	−0.23	0.177	0.371
Disc area (mm^2^)	0.19	0.739	−0.37	0.026	0.133
Cup Volume (mm^3^)	−0.07	0.902	−0.17	0.365	0.794
Average GCC (µm)	−0.47	0.310	−0.21	0.213	0.426
VD RPC Whole Image SV (%)	−0.09	0.873	−0.19	0.288	0.795
VD RPC Inside Disc SV (%)	0.36	0.511	−0.30	0.092	0.079
VD RPC Peripapillary global SV (%)	−0.43	0.428	−0.01	0.972	0.249
VD RPC Peripapillary in the SH SV (%)	−0.58	0.249	−0.06	0.739	0.123
VD RPC Peripapillary in the IH SV (%)	−0.16	0.779	0.09	0.622	0.520
VD RPC Whole Image ALL (%)	−0.23	0.678	−0.20	0.279	0.915
VD RPC Inside Disc ALL (%)	0.37	0.503	−0.22	0.212	0.117
VD RPC Peripapillary global ALL (%)	−0.38	0.483	−0.11	0.533	0.457
VD RPC Peripapillary in the SH ALL (%)	−0.58	0.250	−0.11	0.559	0.155
VD RPC Peripapillary in the IH ALL (%)	−0.23	0.685	0.00	0.997	0.549
VD RPC Peripapillary in the SQ SV (%)	−0.59	0.242	0.12	0.500	0.041 *
VD RPC Peripapillary in the NQ SV (%)	−0.38	0.693	0.05	0.804	0.319
VD RPC Peripapillary in the IQ SV (%)	−0.19	0.740	0.01	0.968	0.612
VD RPC Peripapillary in the TQ (%)	0.28	0.618	−0.09	0.647	0.341
VD Central Macular (%)	−0.33	0.546	−0.19	0.285	0.683

## Data Availability

The data presented in this study are available in the article.

## References

[1] Harris A., Guidoboni G., Siesky B., Mathew S., Verticchio Vercellin A.C., Rowe L., Arciero J. (2020). Ocular blood flow as a clinical observation: Value, limitations and data analysis. Prog. Retin. Eye Res..

[2] Shin J.D., Wolf A.T., Harris A., Verticchio Vercellin A., Siesky B., Rowe L.W., Packles M., Oddone F. (2022). Vascular biomarkers from optical coherence tomography angiography and glaucoma: Where do we stand in 2021?. Acta Ophthalmol..

[3] Miguel A.I.M., Silva A.B., Azevedo L.F. (2019). Diagnostic performance of optical coherence tomography angiography in glaucoma: A systematic review and meta-analysis. Br. J. Ophthalmol..

[4] Lu P., Xiao H., Liang C., Xu Y., Ye D., Huang J. (2020). Quantitative Analysis of Microvasculature in Macular and Peripapillary Regions in Early Primary Open-Angle Glaucoma. Curr. Eye Res..

[5] Rao H.L., Pradhan Z.S., Suh M.H., Moghimi S., Mansouri K., Weinreb R.N. (2020). Optical Coherence Tomography Angiography in Glaucoma. J. Glaucoma.

[6] Chang R., Nelson A.J., LeTran V., Vu B., Burkemper B., Chu Z., Fard A., Kashani A.H., Xu B.Y., Wang R.K. (2019). Systemic Determinants of Peripapillary Vessel Density in Healthy African Americans: The African American Eye Disease Study. Am. J. Ophthalmol..

[7] Miguel A., Silva A., Barbosa-Breda J., Azevedo L., Abdulrahman A., Hereth E., Abegão Pinto L., Lachkar Y., Stalmans I. (2022). OCT-angiography detects longitudinal microvascular changes in glaucoma: A systematic review. Br. J. Ophthalmol..

[8] Koustenis A., Harris A., Gross J., Januleviciene I., Shah A., Siesky B. (2017). Optical coherence tomography angiography: An overview of the technology and an assessment of applications for clinical research. Br. J. Ophthalmol..

[9] Kashani A.H., Chen C.L., Gahm J.K., Zheng F., Richter G.M., Rosenfeld P.J., Shi Y., Wang R.K. (2017). Optical coherence tomography angiography: A comprehensive review of current methods and clinical applications. Prog. Retin. Eye Res..

[10] Siesky B., Harris A., Belamkar A., Zukerman R., Horn A., Verticchio Vercellin A., Mendoza K.A., Sidoti P.A., Oddone F. (2022). Glaucoma Treatment Outcomes in Open Angle Glaucoma Patients of African Descent. J. Glaucoma.

[11] Siesky B., Harris A., Carr J., Verticchio Vercellin A., Hussain R.M., Parekh Hembree P., Wentz S., Isaacs M., Eckert G., Moore N.A. (2016). Reductions in Retrobulbar and Retinal Capillary Blood Flow Strongly Correlate with Changes in Optic Nerve Head and Retinal Morphology Over 4 Years in Open-angle Glaucoma Patients of African Descent Compared with Patients of European Descent. J. Glaucoma.

[12] Gedde S.J., Vinod K., Wright M.M., Muir K.W., Lind J.T., Chen P.P., Li T., Mansberger S.L. (2021). American Academy of Ophthalmology Preferred Practice Pattern Glaucoma Panel. Primary Open-Angle Glaucoma Preferred Practice Pattern^®^. Ophthalmology.

[13] RTVue XR Avanti System (2014). RTVue XR Avanti User Manual.

[14] Siesky B., Harris A., Racette L., Abassi R., Chandrasekhar K., Tobe L.A., Behzadi J., Eckert G., Amireskandari A., Muchnik M. (2015). Differences in ocular blood flow in glaucoma between patients of African and European descent. J. Glaucoma.

[15] Friedman D.S., Wolfs R.C., O’Colmain B.J., Klein B.E., Taylor H.R., West S., Leske M.C., Mitchell P., Congdon N., Kempen J. (2004). Prevalence of open-angle glaucoma among adults in the United States. Arch. Ophthalmol..

[16] U.S. Department of Health and Human Services (HHS). Office of Minority Health Diabetes and African Americans. https://minorityhealth.hhs.gov/omh/browse.aspx?lvl=4&lvlid=18.

[17] Wong D., Chua J., Tan B., Yao X., Chong R., Sng C.C.A., Husain R., Aung T., Garway-Heath D., Schmetterer L. (2021). Combining OCT and OCTA for Focal Structure-Function Modeling in Early Primary Open-Angle Glaucoma. Investig. Ophthalmol. Vis. Sci..

[18] Hammer M., Vilser W., Riemer T., Schweitzer D. (2008). Retinal vessel oximetry-calibration, compensation for vessel diameter and fundus pigmentation and reproducibility. J. Biomed. Opt..

